# Radiomics-Based Method for Predicting the Glioma Subtype as Defined by Tumor Grade, IDH Mutation, and 1p/19q Codeletion

**DOI:** 10.3390/cancers14071778

**Published:** 2022-03-31

**Authors:** Yingping Li, Samy Ammari, Littisha Lawrance, Arnaud Quillent, Tarek Assi, Nathalie Lassau, Emilie Chouzenoux

**Affiliations:** 1 Laboratoire d’Imagerie Biomédicale Multimodale Paris Saclay, BIOMAPS, UMR1281 Inserm, CEA, CNRS, Université Paris-Saclay, 94805 Villejuif, France; samy.ammari@gustaveroussy.fr (S.A.); littisha.lawrance@gustaveroussy.fr (L.L.); nathalie.lassau@gustaveroussy.fr (N.L.); 2 Centre de Vision Numérique, Institut National de Recherche en Informatique et en Automatique (INRIA), Université Paris-Saclay, 91190 Gif-sur-Yvette, France; arnaud.quillent@inria.fr (A.Q.); emilie.chouzenoux@centralesupelec.fr (E.C.); 3 Département d’Imagerie Médicale, Gustave Roussy Cancer Campus Grand Paris, Université Paris-Saclay, 94805 Villejuif, France; 4 Département de Médecine Oncologique, Gustave Roussy Cancer Campus Grand Paris, Université Paris-Saclay, 94805 Villejuif, France; tarek.assi@gustaveroussy.fr

**Keywords:** radiomics, gliomas, glioblastomas, tumor grade, IDH mutation, 1p/19q codeletion

## Abstract

**Simple Summary:**

In 2016, the World Health Organization (WHO) recommended the incorporation of molecular parameters, in addition to histology, for an optimal definition of the central nervous system (CNS) tumors. Gliomas, being among the most common types of CNS tumors, have distinct clinical outcomes and treatment strategies based on different tumor grades, isocitrate dehydrogenase (IDH) mutation, and 1p/19q codeletion statuses. This paper uses radiomics models to noninvasively predict the glioma subtype with clinical Magnetic Resonance Imaging (MRI) images. Different settings in the radiomics pipeline were investigated to achieve optimal performance, together with a better understanding of the exact role of each setting in the model performance. The characteristics of the radiomic features that best distinguish the glioma subtypes were also analyzed. This paper not only provides a radiomics pipeline which works well for predicting the glioma subtype, but it also contributes to the radiomics model development and interpretability.

**Abstract:**

Gliomas are among the most common types of central nervous system (CNS) tumors. A prompt diagnosis of the glioma subtype is crucial to estimate the prognosis and personalize the treatment strategy. The objective of this study was to develop a radiomics pipeline based on the clinical Magnetic Resonance Imaging (MRI) scans to noninvasively predict the glioma subtype, as defined based on the tumor grade, isocitrate dehydrogenase (IDH) mutation status, and 1p/19q codeletion status. A total of 212 patients from the public retrospective The Cancer Genome Atlas Low Grade Glioma (TCGA-LGG) and The Cancer Genome Atlas Glioblastoma Multiforme (TCGA-GBM) datasets were used for the experiments and analyses. Different settings in the radiomics pipeline were investigated to improve the classification, including the Z-score normalization, the feature extraction strategy, the image filter applied to the MRI images, the introduction of clinical information, ComBat harmonization, the classifier chain strategy, etc. Based on numerous experiments, we finally reached an optimal pipeline for classifying the glioma tumors. We then tested this final radiomics pipeline on the hold-out test data with 51 randomly sampled random seeds for reliable and robust conclusions. The results showed that, after tuning the radiomics pipeline, the mean AUC improved from 0.8935 (±0.0351) to 0.9319 (±0.0386), from 0.8676 (±0.0421) to 0.9283 (±0.0333), and from 0.6473 (±0.1074) to 0.8196 (±0.0702) in the test data for predicting the tumor grade, IDH mutation, and 1p/19q codeletion status, respectively. The mean accuracy for predicting the five glioma subtypes also improved from 0.5772 (±0.0816) to 0.6716 (±0.0655). Finally, we analyzed the characteristics of the radiomic features that best distinguished the glioma grade, the IDH mutation, and the 1p/19q codeletion status, respectively. Apart from the promising prediction of the glioma subtype, this study also provides a better understanding of the radiomics model development and interpretability. The results in this paper are replicable with our python codes publicly available in github.

## 1. Introduction

**Figure 1:**
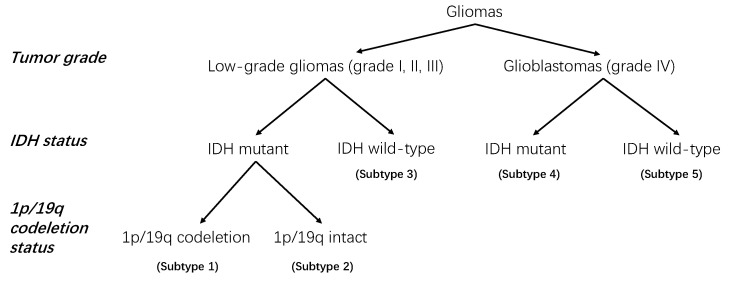
Classification of the glioma tumors used in this paper. This is a simplified version of the classification criteria of gliomas from the 2016 CNS WHO [2]. The subtype numbers 1 to 5 represent the five different glioma subtypes. They can be summarized, respectively, as subtypes “1—LGG, IDH mutant, 1p/19q codeleted”, “2—LGG, IDH mutant, 1p/19q intact”, “3—LGG, IDH wild type”, “4—GBM, IDH mutant”, and “5—GBM, IDH wild type”.

Gliomas are among the most common primary central nervous system (CNS) tumors in the brain [1]. The 2016 World Health Organization Classification of Tumors of the Central Nervous System (2016 CNS WHO) modified the classification criteria of gliomas from its 2007 version, by now defining the glioma entity based on its molecular status and tumor histology [2]. Figure 1 in paper [2] shows a simplified decision tree of the glioma classification based on the 2016 CNS WHO. Apart from the not otherwise specified (NOS) subtype, there are five subtypes of gliomas classified according to tumor histology, isocitrate dehydrogenase (IDH) mutation status, and 1p/19q codeletion status. Astrocytoma, oligoastrocytoma, and oligodendroglioma, which all belong to the low-grade gliomas (LGG, with WHO grades I, II, and III), follow the same classification branch, in contrast to glioblastoma (Grade IV), which follows another. Thus, we propose to simplify the aforementioned criteria by relying on the tumor grade instead of the histology and summarize the classification of gliomas based on tumor grade, IDH mutation, and 1p/19q codeletion status, which yields Figure 1.

The five different subtypes of gliomas (according to different tumor grades, IDH mutation, and 1p/19q codeletion statuses) are characterized by distinct clinical outcomes and, thus, benefit from different treatments. For example, if one patient is diagnosed with low-grade glioma (LGG), then the IDH-mutant and 1p/19q-codeletion status indicates the most favorable prognosis, while IDH mutant without 1p/19q codeletion corresponds to an intermediate prognosis, and IDH wild type leads to the poorest outcomes that are almost similar to the prognosis of glioblastoma [3]. If a patient is diagnosed with glioblastoma, also known as glioblastoma multiforme (GBM), then the IDH-mutant cases usually have better prognoses than those with IDH wild type [4]. Notably, although not considered in this paper, the O6-methylguanine-DNA methyltransferase (MGMT) promoter methylation is also one of the most important biomarkers for glioblastomas. For example, patients with secondary glioblastoma have the best response to temozolomide (standard of care for glioblastoma in the first-line setting [4]) if the tumor is IDH-mutated and MGMT-methylated [5].

Therefore, detecting such molecular signatures and tumor subtypes is crucial for the diagnosis, prognosis, and treatment planing of glioma patients. Stereotactic brain biopsy is the standard of care procedure for the diagnosis of brain tumors. Nevertheless, it is invasive with the risk of inter-reader variability and sampling errors [6]. Therefore, it is important to propose a fast, noninvasive, efficient, and reliable approach for the prediction of tumor grade and molecular characterization in gliomas.

Radiomics is a promising and plausible alternative method, which has become increasingly popular in recent years [7,8,9]. A radiomics-based approach extracts the radiomic features from the clinical medical images, then fits these features and some other clinical information into a machine learning model, aiming to solve a specific medical problem. When genomic data are involved in radiomics, the latter is then termed as radiogenomics. Previous researches have evaluated the role of radiomics in predicting the tumor grade [10,11,12,13,14,15,16,17,18], the IDH mutation status [19,20,21,22,23,24,25,26,27,28,29], the 1p/19q codeletion status [30,31,32,33], or all three glioma labels [34,35].

In this paper, we also used the radiomics method to analyze the tumor grade, IDH mutation, and 1p/19q codeletion status of the gliomas and, thus, to predict the glioma subtype as defined by the 2016 CNS WHO. This task can be regarded as a multilabel classification task (each tumor can have three labels: tumor grade, IDH mutation status, and 1p/19q codeletion status), or a multiclass classification task (five subtypes, as we show in Figure 1). A common method to perform such multilabel or multiclass classification tasks involves converting it into a list of binary classification tasks. Specifically, here, we tackle three binary classification tasks, namely, (1) LGG vs. GBM; (2) IDH mutant vs. IDH wild type; and (3) 1p/19q codeletion vs. 1p/19q intact. Based on the prediction results obtained with these three binary classification tasks, we then predict the tumor subtype based on the criteria in Figure 1. In contrast to the previous publications, we perform a deeper analysis to assess the influence of the various factors in the radiomics pipeline on the classification performance. In detail, we study the impact of the intensity normalization, the radiomics feature extractor, the image filters applied to the medical images before extracting the features, the introduction of the clinical information, the ComBat harmonization method, etc. As the three binary classification tasks may be dependent and correlated, we also linked these tasks into a classifier chain to see whether it would help improve the performances.

## 2. Materials and Methods

### 2.1. Datasets

The public retrospective datasets The Cancer Genome Atlas Glioblastoma Multiforme (TCGA-GBM) [36,37] and The Cancer Genome Atlas Low Grade Glioma (TCGA-LGG) [37,38] were used in our paper. They were used to develop the radiomics models, to predict the tumor grade, IDH mutation status, and 1p/19q codeletion status of the glioma patients, each corresponding to a binary classification task. We introduce hereafter the Magnetic Resonance Imaging (MRI) images and the molecular status of the datasets, and then describe how we clean the data before the analysis.

#### 2.1.1. Clinical MRI Scans

The clinical MRI scans of the TCGA-GBM and TCGA-LGG datasets were downloaded from the Cancer Imaging Archive (TCIA [39], https://www.cancerimagingarchive.net/, accessed on 18 July 2021) platform, which de-identifies and hosts numerous medical images of different cancers for public download purposes. We downloaded the data of 243 glioma patients from the TCIA, including 135 patients (102 train data and 33 test data) from the TCGA-GBM dataset and 108 patients (65 train data and 43 test data) from the TCGA-LGG dataset.

For each patient, four 3D-MRI images representing four different MRI sequences are available, including the native T1-weighted (T1), post-contrast T1-weighted (T1Gd), T2-weighted (T2), and T2 fluid attenuated inversion recovery (T2-FLAIR) volumes. These MRI images were originally collected from multiple institutions, using different MRI scanners and different clinical protocols. Then, before being published in the TCIA platform, these MRI images were preprocessed—they were reoriented to the left posterior superior (LPS) coordinate system, co-registered to the same anatomical template (SRI24 [40]), resampled to a 1 × 1 × 1 mm voxel resolution, and then skull-stripped [37]. Note that, as mentioned in [37], the bias field correction method should not be applied because it may mistake the tumor region for intensity inhomogeneity artifacts and, thus, obliterate the T2-FLAIR signal.

**Figure 2:**
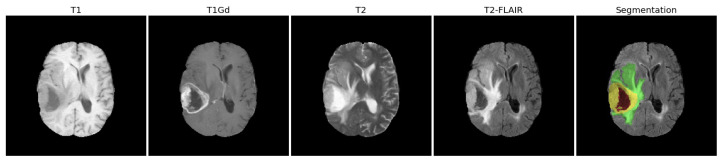
The four MRI sequences (T1, T1Gd, T2, T2-FLAIR) and the corresponding segmentation mask of an example patient “TCGA-02-0047”. The whole tumor (WT) consists of the necrotic part (NCR, red color), the enhancing tumor part (ET, yellow color), and the peritumoral edematous tissue (ED, green color). The tumor core (TC) consists of the necrotic part (NCR, red color) and the enhancing part (ET, yellow color).

Besides the four MRI volumes, the segmentation mask corresponding to the four co-registered MRI scans is also available for each patient. Two different segmentation masks are provided here, (a) with the “_GlistrBoost.nii.gz” suffix representing the computer-aided segmentation masks achieved by GLISTRboost [41]; (b) with the “_GlistrBoost_ManuallyCorrected.nii.gz” suffix, representing the manually corrected segmentation labels, which are corrected on the basis of the automatic segmentation masks by GLISTRboost [37]. In our analysis, we chose the manually corrected segmentation mask when available, otherwise we used the GLISTRboost segmentation mask. We show an example slice of the four MRI volumes and the corresponding segmentation mask of one patient in Figure 2.

Each segmentation mask covers one glioma tumor, with different label values representing different tumor parts, namely, the necrotic/non-enhancing tumor core (NCR or NET, label = 1), the peritumoral edematous/invaded tissue (ED, label = 2), and the GD-enhancing tumor (ET, label = 4) [37,42]. Moreover, the combination of the necrosis (NCR) part and the enhancing tumor (ET) part is named as the tumor core (TC), and the whole tumor (WT) contains the tumor core (TC) and the peritumoral edema (ED) [42]. The segmentation image in Figure 2 shows these subregions of a glioma tumor in different colors, as an example. Specifically, some glioma tumors in our dataset may not have an enhancing tumor (ET) or an edema (ED) zone.

#### 2.1.2. Clinical Information and Molecular Status

For patients involved in the TCGA-GBM and TCGA-LGG datasets, their molecular status (tumor grade, IDH mutation status, 1p/19q codeletion status, etc.) and clinical information (age, sex) were downloaded from The Cancer Genome Atlas (TCGA, https://www.cancer.gov/about-nci/organization/ccg/research/structural-genomics/tcga, accessed on 18 July 2021) platform using TCGAbiolinks and R codes.

#### 2.1.3. Data Cleaning

The downloaded clinical MRI scans, the clinical information (age, sex), and the target variables (the tumor grade, IDH mutation, and 1p/19q codeletion status) can be concatenated by the patient ID. Prior to using the data in radiomics models, for further analysis, we cleaned them as follows:

- Dropped two patients (“TCGA-06-0177” and “TCGA-CS-4941”) because of their very poor image qualities and abnormal image histograms.
- Dropped patient “TCGA-EZ-7265A” because the patient ID could not be found in the TCGA platform.
- Dropped 23 patients who lacked IDH mutation statuses. Among the 23 patients, 22 patients were of the GBM type and 1 was of the LGG type.
- Dropped five patients who lacked the 1p/19q codeletion status. Among the five patients, four patients were GBM with the IDH wild type, one patient was GBM with the IDH mutant.

After dropping these patients, 212 patients were left for our analysis to predict the glioma subtype. We summarize the repartition size of the dataset regarding the tumor grade, IDH mutation status, and 1p/19q codeletion status in Table 1. It can be seen that, in our dataset, most low-grade gliomas were the IDH-mutant, while most glioblastomas were IDH wild type. Only 27 out of the 212 glioma patients were 1p/19q-codeleted, and they were all from the IDH-mutant low-grade gliomas.

The 212 patients were then randomly split into the train and test data, with the ratio of 7:3, in a stratified fashion (stratified by the five glioma subtypes), keeping the same data distributions in the train and test data. The detailed sizes of the train and test data regarding the different target variables are listed in Appendix A.1. The train data were used for the radiomics pipeline tuning, model selection, and hyperparameter tuning with a five-fold cross-validation, while the hold-out test data were used to test the performance of the final model.

### 2.2. Image Preprocessing

As stated previously, the clinical MRI scans have already been preprocessed, including reorientation, co-registration, resampling, and skull-stripping. Li et al. [43] demonstrated that the intensity normalization would help relieve the nonbiological variations at the image level, which were introduced by different MRI scanners or protocols when collecting the images. Thus, we show the image histograms and compare the radiomics model performance before and after applying Z-score intensity normalization in the following section.

### 2.3. Extract Radiomic Features

We extracted 106 radiomic features using the python package Pyradiomics [44] for each three-dimensional volume of interest (VOI), consisting of 14 3D shape-based features, 18 first-order features, 16 gray level run length matrix (GLRLM) features, 16 gray level size zone matrix (GLSZM) features, 14 gray level dependence matrix (GLDM) features, 5 neighbouring gray tone difference matrix (NGTDM) features, and 23 gray level co-occurrence matrix (GLCM) features. We extracted these radiomic features from all four MRI sequences (T1, T1Gd, T2, and T2-FLAIR). Notice that the shape features are the same for these four co-registered MRI scans, so for one VOI, we extracted the shape features only once from the four scans for the same patient.

As shown in Figure 2, the whole tumor consists of different parts of the tumor, including the tumor core (necrosis and enhancing tumor parts) and edema. Thus, the radiomic features could be extracted from five possible subregions, namely, necrosis (NCR), enhancing tumor (ET), edema (ED), tumor core (TC), and whole tumor (WT). However, some tumors do not have ET or ED parts, so instead of extracting radiomic features from ET or ED parts, we used two indicator columns to provide information as to whether ET or ED exist in a glioma tumor. We then compared the classification performances of different feature strategies in the model tuning, Section 3.1.2.

### 2.4. Basic Radiomics Pipeline

In order to predict the glioma subtype, we trained three radiomics models to predict the glioma grade, IDH mutation status, and 1p/19q codeletion status, each corresponding to a binary classification task. First, we used the following basic radiomics pipeline for binary classification as a benchmark, and then gradually tuned the pipeline to improve the classification performance.

(1) Medical images: we used the clinical MRI scans without any intensity normalization or image filters for the radiomics feature extraction.
(2) Feature extraction: for each patient, we extracted the radiomic features from the whole tumor (WT) volume in the four MRI scans (T1, T1Gd, T2, T2-FLAIR).
(3) Feature scaling: we applied a robust scaler [45] to scale features robustly to outliers.
(4) Feature selection: we used the ANOVA F-test as the feature selection method because of its high efficiency and good performance. We set the number of selected features as a hyperparameter to be tuned from set {20,40,60,80,100} by random search and five-fold cross validation. The highly correlated features were not "dropped out", because this step significantly increased the training time without obviously improving the performance in our experiments.
(5) Classifiers: seven classifiers were compared, namely, support vector machine (SVM), perceptron, logistic regression, decision tree, random forest, extra trees, and gradient boosting. The hyperparameters of each classifier were tuned automatically by a random search method (50 parameter settings were randomly sampled) and five-fold cross validation. Some specific hyperparameters were included in this hyperparameter tuning process to avoid overfitting, such as the regularization methods and the regularization parameters used in SVM, perceptron, and logistic regression, and the parameters, such as “max_depth”, “min_samples_split” and “min_samples_leaf” for the tree-based methods. The area under the receiver operating characteristic curve (ROC AUC, or AUC for simplicity) was used as the score metric in the random search. The classifier that achieved the highest mean cross-validation AUC among the seven classifiers was chosen as the final classifier.

### 2.5. Tuning the Radiomics Pipeline

We gradually tuned the basic radiomics pipeline defined above to obtain better classification performance. First, Z-score intensity normalization was applied to the MRI scans to see how it influences the MRI images and the radiomics models. Second, different feature extraction strategies were compared, such as extracting features only from the whole tumor region, or from the necrosis, tumor core, and whole tumor regions, or whether incorporating the ED and ET indicator columns. Third, since there are numerous image filters available in Pyradiomics, including wavelet, Laplacian of Gaussian (LoG), square, square root, logarithm, exponential, gradient, and local binary pattern, we selected the one that performed the best for each task. Fourth, clinical information, such as age and sex, were easy to collect from the patients, so we tested whether to incorporate them into the radiomics models. After that, ComBat [46], a popular harmonization method in radiomics to remove the nonbiological scanner effects, was applied. Then, strategies for data imbalance were applied to deal with the data imbalance present in the 1p/19q codeletion prediction task. Lastly, the prediction of the glioma grade, IDH mutation, and 1p/19q codeletion may be correlated. Thus, inspired by the classifier chain [47], we linked the three classification tasks into a chain, and used the predicted labels of the former classifiers in the chain as additional input for the current classifier, to see whether it could help improve the performance.

## 3. Results

All of the experiments and the computational statistics provided in this section were performed using python codes, available at https://github.com/Yingping-LI/Radiomics (accessed on 29 March 2022) for reproducibility purpose.

### 3.1. Pipeline Tuning

Based on the basic radiomics pipeline defined in Section 2.4, we now discuss how each modification in the radiomics pipeline influences the performance of the classification tasks. All of the random seeds involved in this subsection were set to 2021 to make our results reproducible. Box plots were used for better illustrative and intuitive comparisons among the abundant experiments, where the values in the y-axis represent the AUC values reported on the validation sets in the cross validation process. In each box plot, the median cross-validation AUC is marked by a median line with the accurate value displayed over it, while the mean cross-validation AUC is marked by green triangles. The diamond-shape points located outside the whiskers of the box plot represent the outliers.

#### 3.1.1. Impact of Z-Score Intensity Normalization

**Figure 3:**
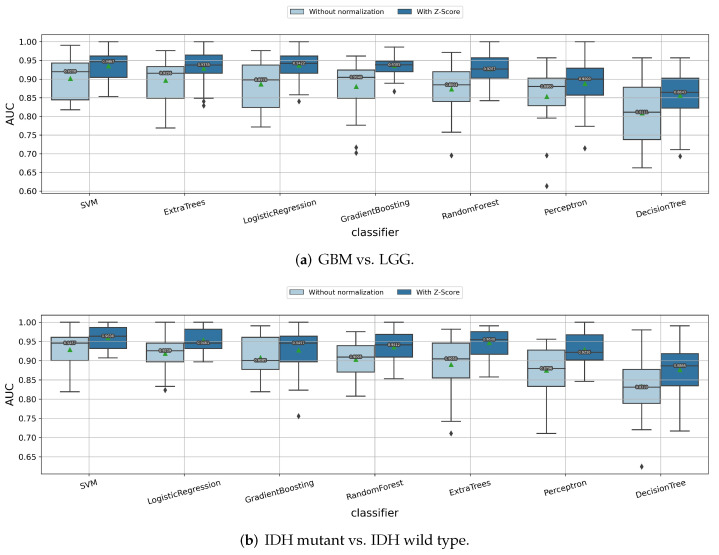

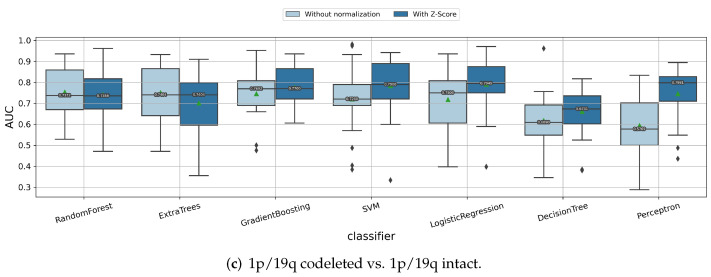
Impact of the Z-score normalization in predicting (**a**) tumor grade, (**b**) IDH mutation status, and (**c**) 1p/19q codeletion status.

**Figure A1:**
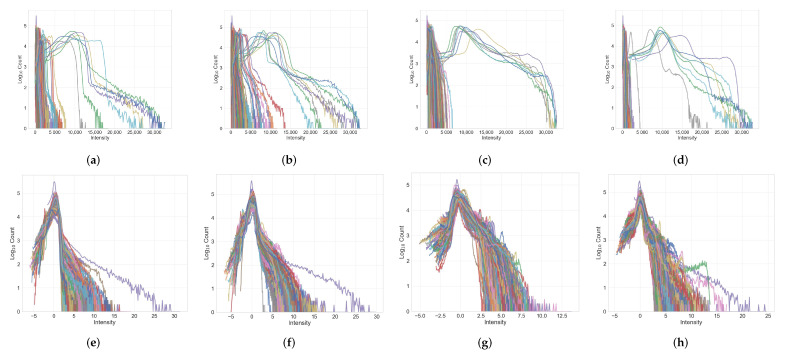
The intensity histograms of the original images (first row) and the Z-score normalized images (second row) for the four sequences of the MRI images. Each colored line corresponds to an image histogram of one preprocessed brain MRI image downloaded from the TCGA-GBM or TCGA-LGG dataset. The subfigures correspond to the image histograms of (**a**) original T1 MRI, (**b**) original T1Gd MRI, (**c**) original T2 MRI, (**d**) original T2-FLAIR MRI, (**e**) Z-score normalized T1 MRI, (**f**) Z-score normalized T1Gd MRI, (**g**) Z-score normalized T2 MRI, and (**h**) Z-score normalized T2-FLAIR MRI images. Obviously, the Z-score normalized images have more consistent image histograms.

We first present the histograms of the MRI images before and after Z-score intensity normalization (Appendix A Figure A1). The Z-score normalization helps make the image histograms more consistent among the patients. We investigate how Z-score normalization influenced the radiomics model performances for predicting the glioma grades and gene statuses. As shown in Figure 3, when predicting the tumor grade, IDH mutation status, and 1p/19q codeletion status, the radiomic features extracted from the Z-score normalized images always had higher median/mean cross-validation AUC values than those from the original images for almost all of the classifiers. This is a strong indicator that the features extracted from the Z-score normalized images have stronger “feature ability”, to predict the glioma subtype. Thus, in the following experiments, we retained Z-score as a preprocessing method applied to MRI images before extracting the radiomics features.

#### 3.1.2. Impact of the Feature Extraction Strategy

**Figure 4:**
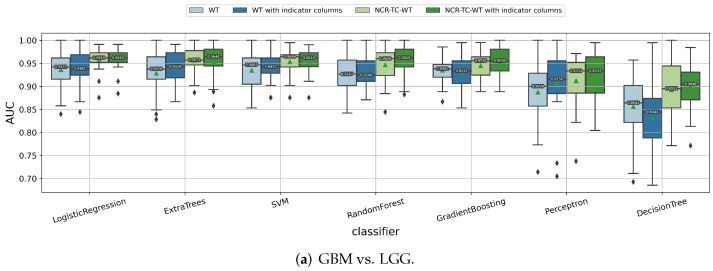

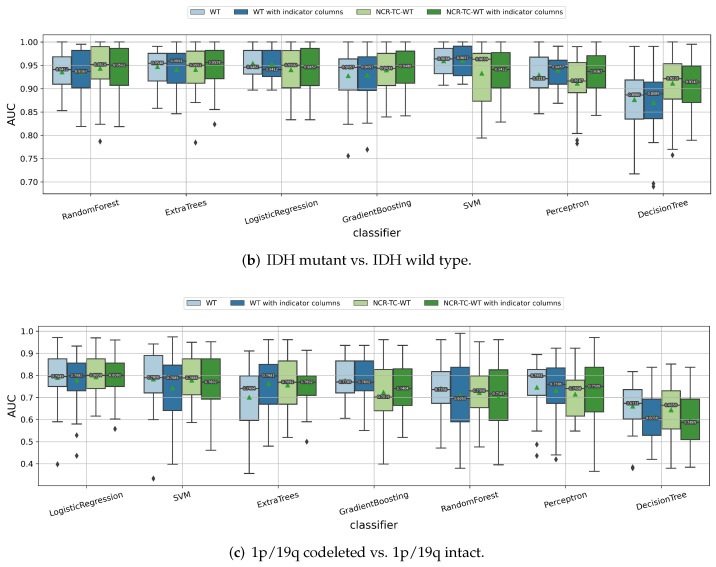
Impact of different feature extraction strategies on predicting (**a**) tumor grade, (**b**) IDH mutation status, and (**c**) 1p/19q codeletion status. WT means using the radiomic features extracted from the whole tumor, and NCR-TC-WT means using features extracted from three subregions including necrosis, tumor core, and whole tumor. “With indicator columns” means two indicator columns are included as additional features to tell whether the glioma tumor has ED or ET parts.

As shown in Figure 4, different feature extraction strategies are compared for their radiomic model performances. Obviously, using radiomic features extracted from three subregions (NCR, TC, WT) has a significant improvement of the median/mean cross-validation AUC values than just using features extracted from the WT region for almost all the classifiers when predicting the tumor grade. When predicting the IDH mutation and 1p/19q codeletion status, incorporating the additional NCR and TC parts for extracting radiomic features does not significantly influence the prediction performances. Thus, to keep consistent for the three binary classification tasks, we chose to use the radiomic features extracted from NCR, TC, and WT parts, with two indicator columns telling whether a glioma tumor has ED or ET parts, so as to include more information without damaging the performances.

To conclude, for each patient, 1146 radiomic features (42 shape features, 216 first-order features, and 888 texture features) and 2 additional indicator features were extracted and fitted into the proposed radiomics pipeline, which included the ANOVA F-test feature selection.

#### 3.1.3. Impact of Image Filters

**Figure 5:**
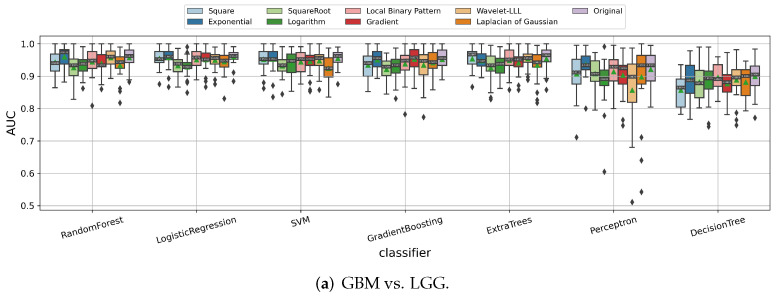

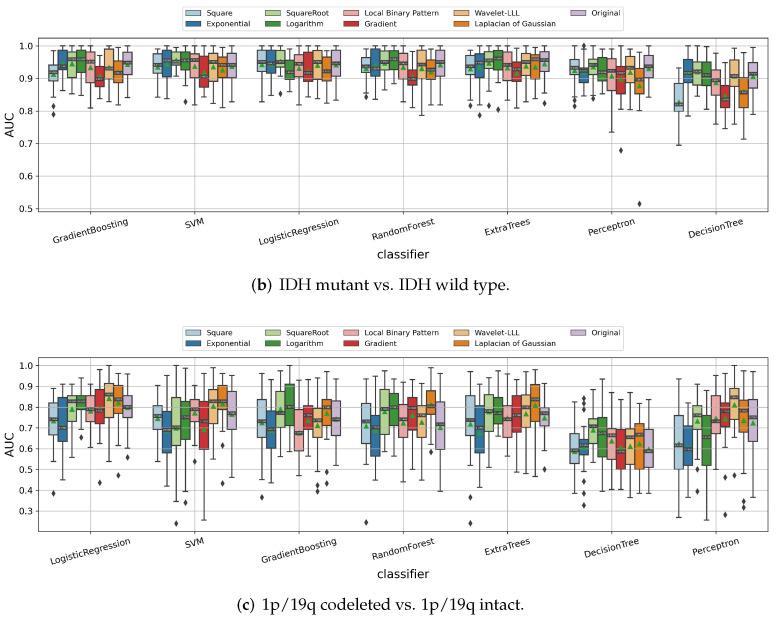
Impact of different image filters for predicting (**a**) tumor grade, (**b**) IDH mutation status, and (**c**) 1p/19q codeletion status of the gliomas. Notably, the wavelet image filter has eight decompositions defined by using different high or low pass filters in each dimension. We chose to display “Wavelet-LLL” here because of its best performance compared to the other wavelet filters in our experiments.

In the above experiments, we used the MRI images without any image filters applied before extracting the radiomic features. We tested all of the image filters available in Pyradiomics to see how they could affect the prediction of the tumor grade, IDH mutation, and 1p/19q codeletion status of the gliomas. As shown in Figure 5, the image filters have an obvious impact on the radiomics model performances. Thus, for each binary classification task, we chose the one that achieved the relatively best performances for almost all of the classifiers, namely, the “original” image filter for predicting tumor grade, “square root” for predicting IDH mutation status, and “Laplacian of Gaussian (LoG)” for predicting 1p/19q codeletion status.

#### 3.1.4. Impact of the Age and Sex Information

**Figure 6:**
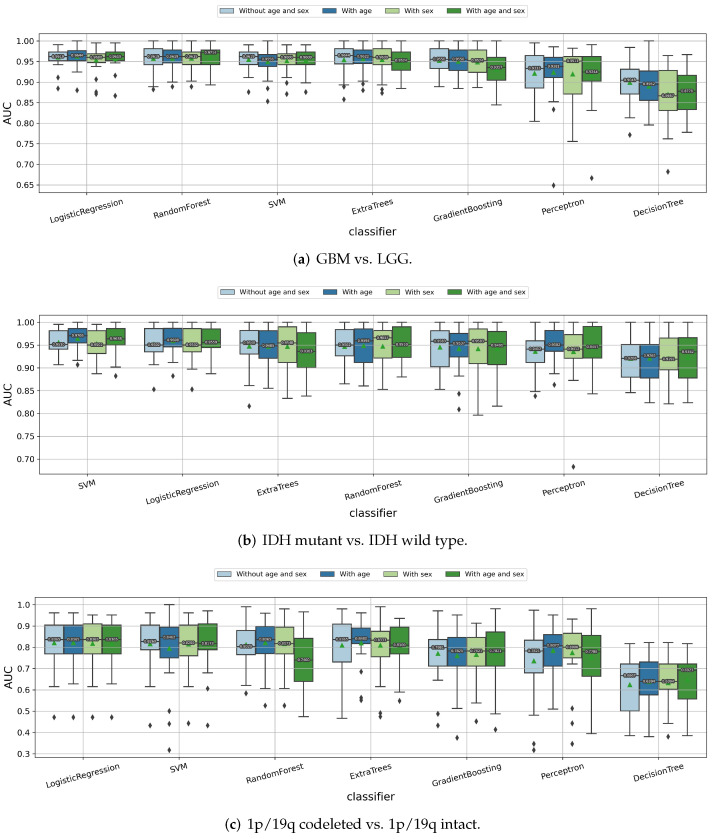
Impact of incorporating the age and sex information as additional features to predict (**a**) tumor grade, (**b**) IDH mutation status, and (**c**) 1p/19q codeletion status of the gliomas.

Apart from the radiomic features extracted from the clinical MRI scans, other clinical information, such as age and sex, were easy to collect from the patients. Thus, we also incorporated this information in the radiomics models to see whether it improved the performances or not. As shown in Figure 6, the age information helps to improve the prediction of the IDH mutation status slightly for almost all of the classifiers, but it does not have a clear influence on the prediction of the tumor grade and 1p/19q codeletion status. Besides the age information, adding the sex feature does not further improve the prediction performances. Hence, we did not incorporate the sex information but only the patient age, as an additional feature in our radiomics models to predict the IDH mutation status. The age information was also included when predicting the tumor grade and 1p/19q codeletion status, for better consistency among the three classification tasks.

#### 3.1.5. Impact of ComBat Harmonization

**Figure 7:**
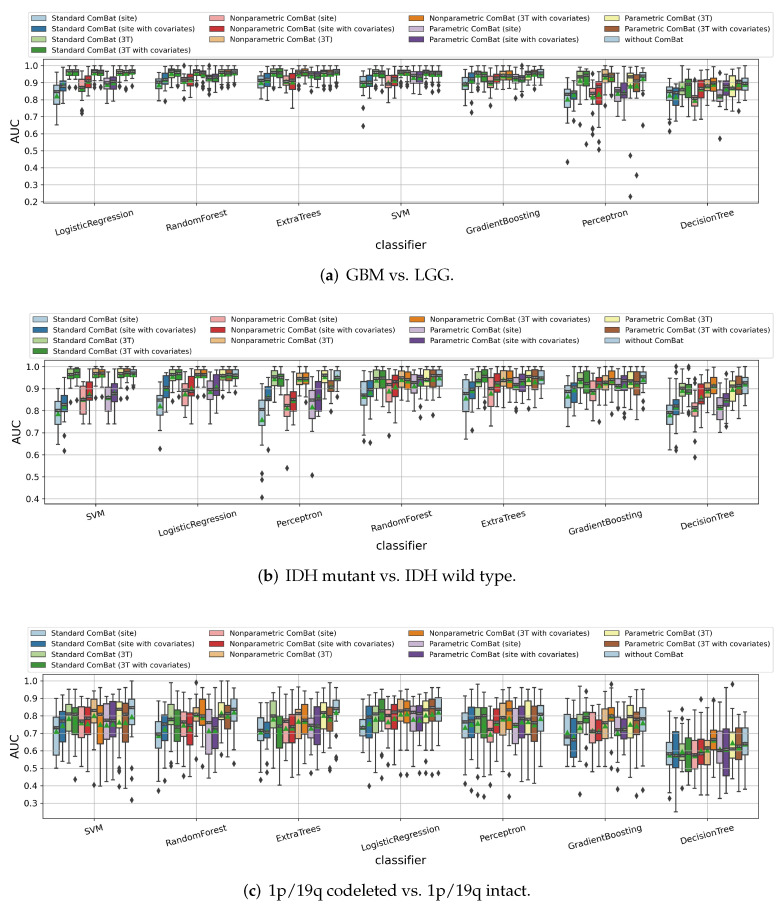
Impact of ComBat harmonization on predicting (**a**) tumor grade, (**b**) IDH mutation status, and (**c**) 1p/19q codeletion status of the gliomas. Here “site” corresponds to using the institution/site label as the scanner setting label during harmonization, while “3T” represents using the magnetic field strength (1.5T or 3T) as a scanner setting label. “With covariates” means keeping the age and sex information during the harmonization process.

The TCGA-GBM and TCGA-LGG datasets used in our paper include patients collected from different institutions with different image scanners or clinical protocols; thus, the nonbiological scanner effects might be introduced. We applied Z-score normalization on the MRI images as a preprocessing step; however, according to the conclusion of our recent study [43], only applying the intensity normalization might not be enough for radiomics models. Thus, we tested the ComBat method to see whether it helped improve the feature ability of the radiomic models. As displayed in Figure 7, ComBat does not help in any of the three binary classification tasks. None of the ComBat variants (namely, standard ComBat without using empirical Bayes (EB), parametric ComBat using parametric EB, and nonparametric ComBat using nonparametric EB), appeared to help improve the median/mean cross-validation AUC values significantly, whether using “magnetic field strength” or “site label” as scanner setting labels, or keeping the age and sex information during harmonization. Thus, we decided not to use a ComBat method in the following experiments.

#### 3.1.6. Impact of Data Imbalance Strategy

**Figure 8:**
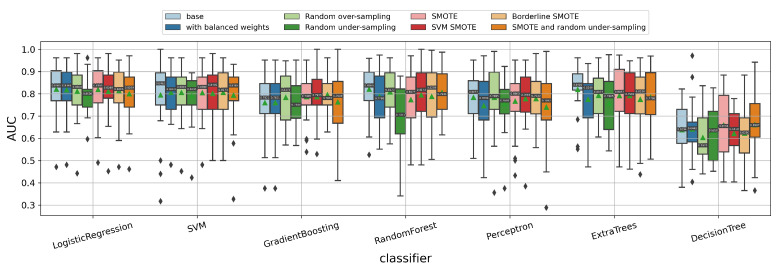
Impact of further data imbalance strategies on predicting the 1p/19q codeletion status of the gliomas. Here, the possible regularization was already applied for each classifier, so “base” means not further using any other data imbalance strategies. “SMOTE and random under-sampling” represents the combination of SMOTE and random under-sampling method.

When predicting the 1p/19q codeletion status, the data are highly imbalanced (27 1p/19q codeleted patients vs. 185 1p/19q intact patients). When tuning the hyperparameters for the classifiers using the random search method, we already added the possible regularization parameters. Here, we further applied some other data imbalance strategies, such as setting the class weights or using data sampling-based methods (random under-sampling/over-sampling, SMOTE [48], SVM-SMOTE [49], Borderline-SMOTE [50], etc.) to deal with the data imbalance. The experiment results are shown in Figure 8. Surprisingly, these data imbalance methods did not further improve the prediction of the 1p/19q codeletion status significantly in our dataset.

#### 3.1.7. Impact of Using Classifier Chain Idea

**Figure 9:**
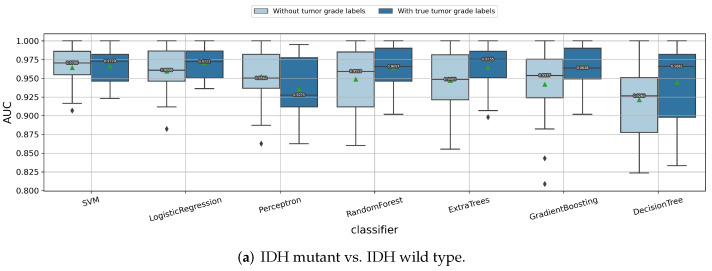

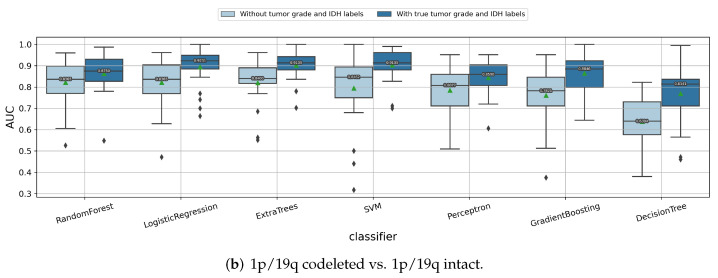
Comparison of whether it helps to improve the performances by using the classifier chain idea, with the true labels of previous classifiers given. (**a**) Predicting IDH mutation status of the gliomas; (**b**) predicting 1p/19q codeletion status of the gliomas.

In the above experiments, we considered the problem of classifying the glioma subtypes as three independent binary classification tasks. However, the three labels we are predicting may be highly correlated. For example, most of the LGG tumors are IDH mutants and most of the GBM tumors are IDH wild type. Therefore, we now consider using the classifier chain idea and linking these three binary classification tasks as a chain, namely, (1) predicting tumor grade → (2) predicting IDH mutation status → (3) predicting 1p/19q codeletion status. When predicting the current label, the results of all the previous classifiers in the chain will be added as additional features. First, we compared if the performance would improve if the true labels of the former classifiers were given. That is, (1) when predicting the IDH mutation status, the true tumor grade is given; and (2) when predicting 1p/19q codeletion status, both the true tumor grade and true IDH mutation status are given. The experiment results in Figure 9 show that, if the true label of the tumor grade and IDH mutation status are given, then there will be a significant improvement of the median/mean cross-validation AUC value for predicting the 1p/19q codeletion status. Similarly, if the true tumor grade is known, then the median/mean cross-validation AUC value for predicting the IDH mutation status also improves, obviously. However, in most cases, both the tumor grade and IDH mutation status are unknown and needed to be predicted. Therefore, in the following experiments, we retained the predicted labels of the former classifiers instead.

### 3.2. Summarized Pipeline

**Figure 10:**
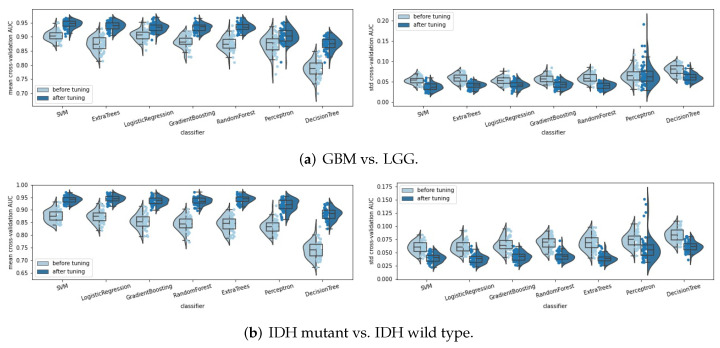

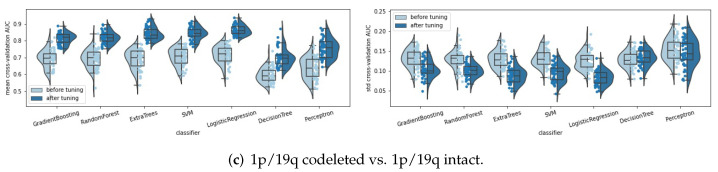
Distribution (displayed by violin plot, box plot, and strip plot) of the mean and STD cross-validation AUC values before and after the pipeline tuning process, for predicting (**a**) the tumor grade, (**b**) IDH mutation status, and (**c**) 1p/19q codeletion status of the gliomas. Each reported point corresponds to a sample random run within 51 random seeds.

In the above subsection, we tuned the settings in the radiomics pipeline. In this subsection, we will summarize the final fine-tuned radiomics pipeline for classifying the gliomas. Notably, the analyses and conclusions in Section 3.1 are based on the fixed random seed 2021. When tuning the settings in the pipeline, we looked at the overall performances of all seven classifiers to avoid a “lucky” choice. Now, after fine-tuning the settings in the radiomics pipeline, we used more random seeds to test the robustness of our fine-tuned radiomics pipeline. A total of 50 integers in (0, 5000) were randomly sampled with the fixed random seed 2021 to compose a set of random seeds. By splitting the train/test data and fitting the radiomics pipeline with these 51 random seeds (including 2021), we achieved 51 mean (standard deviation) cross-validation AUC values, each corresponding to an experiment with a chosen fixed random seed. We compared the 51 mean (standard deviation) cross-validation AUC values before and after the pipeline tuning process and display their distributions by a violin plot in Figure 10. Obviously, by tuning the settings in the radiomics pipeline, the mean cross-validation AUC improved significantly, and the standard deviation of the cross-validation AUC decreased, for almost all seven classifiers and for all three binary classification tasks. This indicates that our pipeline tuning process helps to improve the classification performance significantly and robustly with the obvious higher mean and lower standard deviation of the cross-validation AUC, and is not just a "lucky choice" of a fixed random seed.

We summarized the performance of the seven classifiers on 51 independent random runs, to assess the robustness and efficacy of our classifiers for each task. The average and the 95% confidence interval (CI) of the mean and standard deviation (STD) cross-validation AUC values computed over 51 random seeds before and after the pipeline tuning process are listed in Table 2. From the table, we can easily conclude that, after tuning the settings in the pipeline, SVM achieved the best performance for predicting the tumor grade, with an average “mean cross-validation AUC” of 0.9443 (95% CI: [0.9403, 0.9483]) and an average “STD cross-validation AUC” of 0.0372 (95% CI: [0.0348, 0.0395]). When predicting the IDH mutation labels, logistic regression works the best, with an average “mean cross-validation AUC” of 0.9445 (95% CI: [0.9408, 0.9481]) and an average “STD cross-validation AUC” of 0.0379 (95% CI: [0.0356, 0.0403]). Logistic regression also outperforms the other classifiers when predicting the 1p/19q codeletion status, with an average “mean cross-validation AUC” of 0.8631 (95% CI: [0.8553, 0.8710]) and an average “STD cross validation AUC” of 0.0831 (95% CI: [0.0783, 0.0879]). Thus, in our final radiomics pipeline, SVM was used for predicting the tumor grade, and logistic regression was chosen for predicting both the IDH mutation and 1p/19q codeletion labels.

**Figure 11:**
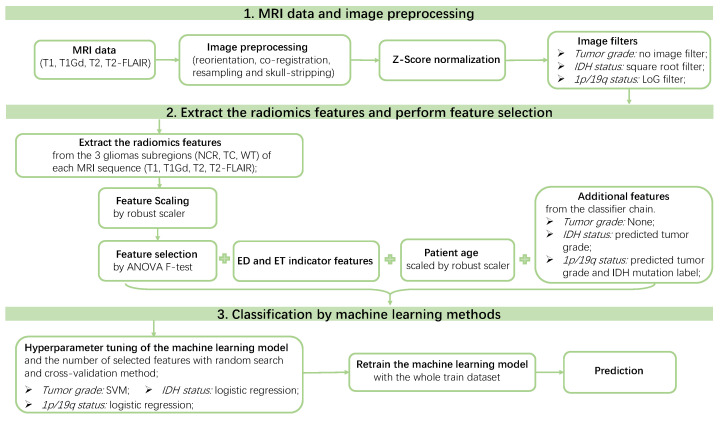
Final radiomics pipeline for predicting each binary classification label (tumor grade, IDH mutation status, and 1p/19q codeletion status) of the gliomas tumor. During the hyperparameter tuning process, the mean cross-validation AUC is used as the evaluation metric.

To conclude, we summarize our final radiomics pipeline for predicting each glioma label (tumor grade, IDH mutation, and 1p/19q codeletion) in Figure 11. In detail, after we obtained the preprocessed MRI data (reorientation, co-registration, resampling, and skull-stripping), Z-score normalization was further applied to normalize the image intensities. Then different image filters were applied to obtain better feature capabilities for each binary classification task. In other words, the square root image filter is applied for predicting IDH mutation status, Laplacian of Gaussian (LoG) image filter was applied to predict 1p/19q codeletion, and the original image, without any image filters, was used to predict the tumor grade. Then 1146 radiomic features were extracted from the processed MRI scans, specifically from the three subregions (including necrosis, tumor core, and whole tumor) of all four processed MRI sequences (T1, T1Gd, T2, and T2-FLAIR). The extracted radiomic features were then scaled by robust scaling and selected using the ANOVA F-test to select the most important features for each task. The selected radiomic features, as well as other features, such as ED/ET indicator features, the scaled patient age, and the predicted labels of the previous classifiers in the classifier chain, were fitted into the machine learning model to tune the hyperparameters of the classifier with random search and the cross-validation method. The number of selected radiomic features were also tuned {20,40,60,80,100} in this process. The model with the hyperparameters corresponding to the highest mean cross-validation AUC were saved and then retrained with the whole train dataset before making the prediction on the test data.

### 3.3. Predict Glioma Subtype by the Final Pipeline

In Section 3.2, we summarize the final radiomics pipeline for predicting each label (tumor grade, IDH mutation status, 1p/19q codeletion status) of the glioma tumor (Figure 11). We used this pipeline and report the prediction performances on the hold-out test dataset, also based on the 51 random seeds for a robust analysis. Moreover, the performance of the basic radiomics pipeline (Section 2.4) without our pipeline tuning process is also listed as a comparison. As shown in Table 3, our final radiomics pipeline outperforms the basic radiomics pipeline significantly, indicating the great significance of our pipeline tuning process. In detail, the mean AUC on the hold-out test data computed on 51 random runs improved significantly, from 0.8935 (±0.0351) to 0.9319 (±0.0386) for predicting the tumor grade, from 0.8676 (±0.0421) to 0.9283 (±0.0333) for predicting the IDH mutation status, and from 0.6473 (±0.1074) to 0.8196 (±0.0702) for predicting the 1p/19q codeletion status. Meanwhile, the mean accuracy also had obvious improvement; that is, from 0.8171 (±0.0442) to 0.8453 (±0.0691), from 0.7840 (±0.0636) to 0.8560 (±0.0333), from 0.7209 (±0.2250) to 0.8499 (±0.0351), for predicting the glioma grade, the IDH mutation status, and the 1p/19q codeletion status, respectively.

Given the predicted labels of the glioma grade, IDH mutation and 1p/19q codeletion status, we could then convert the three predicted glioma labels to the prediction of the five glioma subtypes, according to the simplified criteria of 2016 CNS WHO in Figure 1. On the hold-out test data, compared to the mean accuracy of 0.5772 (±0.0816) by the basic radiomics pipeline, we achieved a mean accuracy of 0.6716 (±0.0655) using our final fine-tuned radiomics pipeline for predicting the five gliomas subtypes.

**Figure 12:**
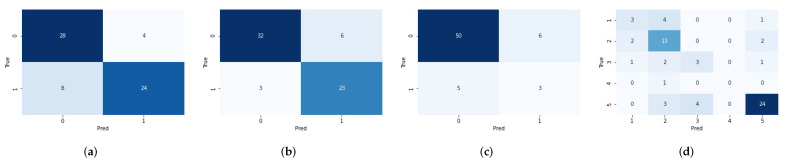
The confusion matrices on the hold-out test data for predicting (**a**) GBM vs. LGG, (**b**) IDH mutant vs. IDH wild type, (**c**) 1p/19q codeleted vs. 1p/19q intact, and (**d**) the five glioma subtypes defined by the 2016 CNS WHO. Random seed was fixed as 4442 because of its median prediction accuracy of the glioma subtype (accuracy = 0.6719) among the 51 random seeds. Here, the accuracy for predicting the tumor grade, IDH mutation, and 1p/19q codeletion was 0.8125, 0.8594, and 0.8281, respectively.

Figure 12 shows the confusion matrices for predicting the tumor grade, IDH mutation status, 1p/19q codeletion status, and the glioma subtype on the hold-out test data using our final radiomics pipeline, with a fixed random seed 4442 as an example for its median prediction accuracy of the glioma subtypes (0.6719) among the 51 random seeds.

As shown in Figure 12d, predicting subtype “2—LGG, IDH mutant, 1p/19q intact” and “5—GBM, IDH wild type” are relatively easy tasks when compared to predicting the other subtypes. We computed the average (STD) of the F1-score for the prediction performance on the hold-out test data for each of the 5 glioma subtypes, averaged on the 51 fixed random seeds. We achieved an average (STD) F1-score of 0.3503 (±0.1717) for predicting subtype “1—LGG, IDH mutant, 1p/19q codeleted”, 0.6688 (±0.1247) for subtype “2—LGG, IDH mutant, 1p/19q intact”, 0.2018 (±0.1550) for subtype “3—LGG, IDH wild type”, 0.0203 (±0.0872) for subtype “4—GBM, IDH mutant”, and 0.8454 (±0.0468) for subtype “5—GBM, IDH wild type”. Note that we obtained a rather low average F1-score of 0.0203 for subtype “4—GBM, IDH mutant”, mainly because we only had three samples of this type for training and one sample for the test.

### 3.4. Interpretability of the Radiomics Models

**Figure 13:**
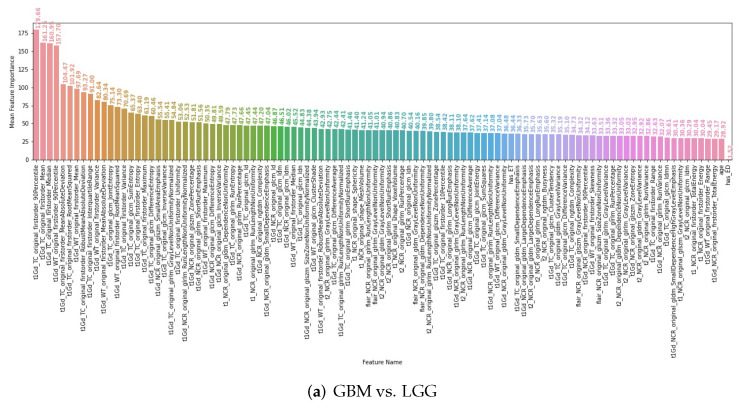

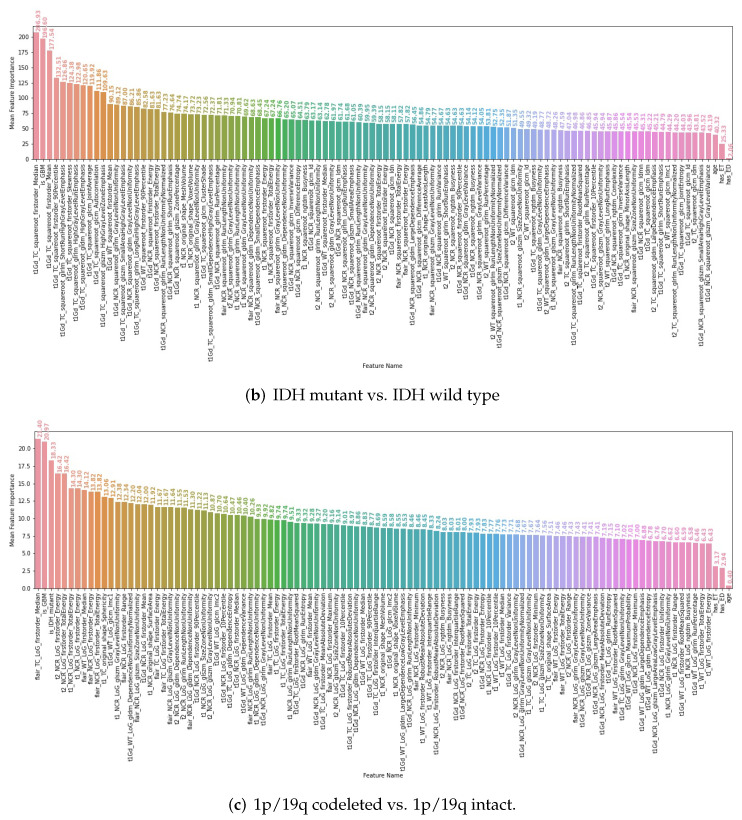
The non-radiomic features and the first 100 radiomic features with the highest average feature importance calculated by the ANOVA F-value for predicting (**a**) tumor grade, (**b**) IDH mutation status, and (**c**) 1p/19q codeletion status. The feature importance annotated on each feature bar refers to the mean feature importance averaged on the 51 random seeds.

To better understand which features distinguished the glioma grade, IDH mutation status, and 1p/19q codeletion status, respectively, we checked the radiomic features extracted by our final radiomics pipeline and the corresponding feature importance calculated by the ANOVA F-value for each task. To avoid the bias caused by a single chosen random seed, for each feature, we averaged their feature importance calculated by the experiments using the 51 random seeds. In Figure 13, we see the first 100 radiomic features with the highest average feature importance and other non-radiomic features, such as age and ED/ET indicator features. Obviously, the predicted tumor grade is an important feature for predicting the IDH mutation status (Figure 13b), while the predicted tumor grade and IDH mutation status are of high feature importance to predict 1p/19q codeletion status (Figure 13c). Moreover, when predicting the tumor grade and IDH mutation status, compared to the ET indicator feature (“has_ET”), the ED indicator feature (“has_ED”) has a very low average feature importance, meaning that the existence of edema is not as related to the tumor grade or the IDH mutation status. Meanwhile, the patient age provides some information for predicting the tumor grade and IDH mutation status, but not for predicting the 1p/19q codeletion status.

**Figure 14:**
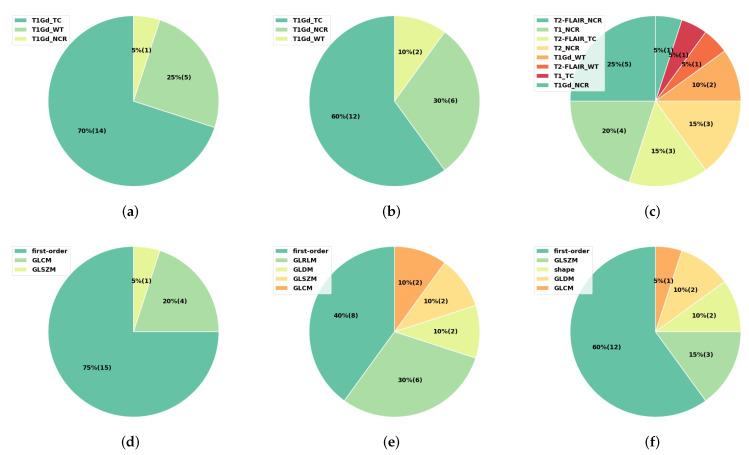
Statistics of the first 20 radiomic features with the highest average feature importance calculated by ANOVA F-value and averaged on 51 random seeds. The first row shows the statistics of the MRI sequences and tumor subregions (**a**) for predicting tumor grade, (**b**) for predicting IDH mutation status, and (**c**) for predicting 1p/19q codeletion status. The second row shows the statistics of the feature types (**d**) for predicting tumor grade, (**e**) for predicting IDH mutation status, and (**f**) for predicting the 1p/19q codeletion status, respectively. The values in the brackets represent the number of features.

For the radiomic features that have the highest average feature importance, we further analyzed their MRI sequences, tumor subregions, and feature types. Here, we performed the statistics on the first 20 important radiomic features to emphasize the most important ones. As the statistics show in Figure 14, the first 20 radiomic features were extracted from the T1Gd sequence for both the tumor grade (Figure 14a) and the IDH mutation (Figure 14b) prediction tasks, while the first 20 important radiomic features for predicting the 1p/19q codeletion were from the four MRI sequences, with 9 out of 20 from the T2-FLAIR scans (Figure 14c). Moreover, the features extracted for predicting the tumor grade were mostly from the tumor core and the whole tumor region (Figure 14a), while the features for predicting the IDH mutation status were mostly from the tumor core and the necrosis region (Figure 14b). For predicting the 1p/19q codeletion status, the first 20 important radiomic features were from the necrosis (13 features), the tumor core (4 features), and the whole tumor (3 features), as displayed in Figure 14c.

Regarding the feature types, among the first 20 radiomic features for predicting the glioma grade, most of them were first-order features (Figure 14d). When predicting the IDH mutation status, the first-order features and other features, especially the GLRLM features, constituted the best 20 radiomic features (Figure 14e). Twelve out of the twenty radiomic features were first-order features when predicting the 1p/19q codeletion status, while the others were from texture feature types, such as GLSZM and GLDM (Figure 14f).

To conclude, when predicting the glioma grade, the most important features are the first-order features extracted from the tumor core and the whole tumor region in the original T1Gd images. The first-order features and GLRLM texture features from the tumor core and necrosis regions in the filtered T1Gd MRI images by square root, as well as the predicted tumor grade, constitute the most important features for predicting IDH mutation status. When predicting the 1p/19q codeletion status, apart from the predicted tumor grade and IDH mutation labels, the first-order features and other texture features, such as GLSZM and GLDM, are of high importance.

## 4. Discussion

In this paper, we investigated the use of radiomics models, a noninvasive method, in predicting the glioma subtype, based on the 2016 CNS WHO. The five subtypes of the gliomas are related to three gliomas features, namely, the tumor grade, the IDH mutation status, and the 1p/19q codeletion status. Thus, instead of taking the problem as a multilabel or multiclass classification problem, we converted it into three binary classification tasks. For each binary classification task, we started with a basic radiomics pipeline and then gradually tuned the settings, with the aim to obtain the possible best classification performance, along with a deeper understanding as to how each modification influences the radiomics model performance. Finally, we arrived at an optimal pipeline and summarized it as a final radiomics pipeline for classifying the glioma tumors. A total of 212 patients from the public retrospective TCGA-LGG and TCGA-GBM datasets were used, so all of the experiment results and analyses in our study are replicable with publicly available codes.

When tuning the settings in the radiomics pipeline, we acquired some interesting findings. First, Z-score normalization not only renders the MRI scans as more comparable among the patients, but it also significantly improves the classification performances for all three binary classification tasks. Second, the image filter applied to the MRI scans before extracting the radiomic features has an obvious impact on the classification performances. In our cases, we chose the relatively best one for each task, namely, the original image without any filters for predicting tumor grade, the square root filter for predicting IDH mutation, and the Laplacian of Gaussian filter for predicting the 1p/19q codeletion status. Third, ComBat harmonization did not help improve the performance of any of the three binary classification tasks in our study. This was not surprising, as Orlhac et al. [51] reported that, among the 51 papers published since 2017 using ComBat harmonization, although 41% reported better performances after using ComBat, there were still 18% that did not observe any benefit in using ComBat; the other 41% presented the results with ComBat harmonization but without reporting the results before ComBat. Moreover, by linking the three binary classification tasks into a chain, we found that, if the true glioma grade is given as an additional feature, then the prediction of IDH status improves significantly. Similarly, there is a significant improvement for predicting 1p/19q codeletion status if the true glioma grade and IDH mutation status are added as additional features. In real practice, the glioma grade and IDH mutation labels are possibly unknown, so we used their predicted labels from our trained radiomics models instead.

Remarkably, after the pipeline tuning process, the prediction of the glioma subtype improved significantly. In detail, on the hold-out data, which is never seen during any pipeline tuning, model selection, or hyperparameter tuning processes, the mean AUC, averaged on the results of multiple random seeds, improves from 0.8935 (±0.0351) to 0.9319 (±0.0386), from 0.8676 (±0.0421) to 0.9283 (±0.0333), and from 0.6473 (±0.1074) to 0.8196 (±0.0702) for predicting the tumor grade, IDH mutation, and 1p/19q codeletion status, respectively. Meanwhile, the mean accuracy also improves, from 0.8171 (±0.0442) to 0.8453 (±0.0691), from 0.7840 (±0.0636) to 0.8560 (±0.0333), from 0.7209 (±0.2250) to 0.8499 (±0.0351), and from 0.5772 (±0.0816) to 0.6716 (±0.0655) for predicting the glioma grade, the IDH mutation status, the 1p/19q codeletion status, and the five glioma subtypes, respectively.

To better interpret the radiomics model, we also analyzed the characteristics of the first 20 radiomic features with the highest average feature importance calculated by the ANOVA F-value. The first-order features extracted from the tumor core and the whole tumor region in the original T1Gd MRI scan can significantly depict the differences in the tumor grade. For predicting the IDH mutation status, the predicted tumor grade, as well as the first-order features and GLRLM texture features from the tumor core and necrosis regions in the T1Gd MRI scans with the square root image filter, are the most important features. For predicting the 1p/19q codeletion status, apart from the predicted tumor grade and IDH mutation labels, the first-order features and other features extracted from the LoG-filtered MRI images are of high importance.

In summary, this paper provides a promising tool for the prediction of the glioma subtype as well as a better understanding of the radiomics model development and the feature interpretability. However, this paper also has some limitations. First, we only used the public retrospective datasets TCGA-GBM and TCGA-LGG in this study. The analyses and conclusions need to be further confirmed on other possible independent datasets. Second, the radiomics model we developed is not an end-to-end manner because the segmentation masks should be provided for the new patients. The automatic segmentation can be achieved by training a neural network, such as nnU-Net [52] with the public dataset of the brain tumor segmentation (BraTS) challenge. The BraTS 2021 dataset [42] consists of 2000 patients from multiple institutions, corresponding to 8000 pre-operative multiparametric MRI scans (T1, T1Gd, T2, T2-FLAIR). We did not use the predicted segmentation labels from the nnU-Net trained with the BraTS 2021 dataset, because TCGA-GBM and TCGA-LGG are part of the BraTS2021 data and cannot be identified from the renamed patient IDs in BraTS 2021 (risking data leakage). We mention here that, although most of the segmentation masks used in our paper (downloaded from TCIA platform) have already been manually corrected based on the automatic segmentation masks achieved by GLISTRboost software, there is still room for improvement, especially when segmenting the necrosis and enhancing tumor parts. Third, the MRI scans downloaded from TCGA-LGG and TCGA-GBM datasets have already been preprocessed, including reorientation, co-registration, resampling, and skull-stripping, so the same preprocessing steps should be performed for the new patients when using our radiomics pipeline. Finally, even though a promising performance in the prediction of the glioma grade and IDH mutation status was achieved, more efforts can be invested (through larger investigations in the future) to improve the prediction of the 1p/19q codeletion status.

## 5. Conclusions

In this paper, we investigated using the radiomics models to noninvasively predict the gliomas subtype as defined by the tumor grade, IDH mutation, and 1p/19q codeletion status. Several investigations were conducted on the public retrospective TCGA-GBM and TCGA-LGG datasets to evaluate the impacts of different settings in the radiomics pipeline on the classification performance, such as Z-score normalization, feature extraction strategy, image filters, ComBat harmonization, classifier chain strategy, etc. After tuning these settings, a final radiomics pipeline for the prediction of the glioma subtype was proposed. Furthermore, the characteristics of the various radiomic features were analyzed to tell which features distinguished the most for predicting the glioma grade, IDH mutation, and 1p/19q codeletion status, respectively.

## Figures and Tables

**Table 1:** Summary of the repartition size of the dataset regarding the tumor grade, IDH mutation status, and 1p/19q codeletion status. Each value represents the number of patients of that glioma type in our dataset.

	IDH Mutant		IDH Wild Type		All
	1p/19q Codeletion	1p/19q Intact	1p/19q Codeletion	1p/19q Intact	
**GBM**	0	4	0	103	107
**LGG**	27	57	0	21	105
**All**	27	61	0	124	212

**Table 2:** Mean and standard deviation (STD) of the cross-validation (CV) AUC values during the cross-validation process for each classifier, before and after the pipeline tuning process. The table reports the average and 95% confidence interval (in square brackets), computed over 51 random realizations, of the mean/std cross-validation AUC for predicting the glioma grade, IDH mutation status, and 1p/19q codeletion status. The best performance for each task is marked in bold italics.

Classifier	Description	Tumor Grade		IDH Mutation Status		1p/19q Codeletion Status	
		Mean CV AUC	Std CV AUC	Mean CV AUC	Std CV AUC	Mean CV AUC	Std CV AUC
**SVM**	**Before tuning**	0.9039 [0.8991, 0.9087]	0.0534 [0.0511, 0.0556]	0.8769 [0.8703, 0.8834]	0.0601 [0.0572, 0.0631]	0.7048 [0.6906, 0.7190]	0.1317 [0.1260, 0.1373]
	**After tuning**	***0.9443*** ***[0.9403, 0.9483]***	***0.0372*** ***[0.0348, 0.0395]***	0.9407 [0.9372, 0.9443]	0.0398 [0.0376, 0.0421]	0.8443 [0.8343, 0.8543]	0.0945 [0.0887, 0.1003]
**Perceptron**	**Before tuning**	0.8723 [0.8632, 0.8813]	0.0659 [0.0614, 0.0704]	0.8328 [0.8256, 0.8400]	0.0743 [0.0705, 0.0782]	0.6370 [0.6198, 0.6541]	0.1531 [0.1456, 0.1607]
	**After tuning**	0.9032 [0.8961, 0.9103]	0.0686 [0.0605, 0.0768]	0.9189 [0.9123, 0.9256]	0.0587 [0.0517, 0.0658]	0.7536 [0.7384, 0.7688]	0.1450 [0.1358, 0.1543]
**Logistic Regression**	**Before tuning**	0.9048 [0.8994, 0.9102]	0.0522 [0.0498, 0.0546]	0.8732 [0.8666, 0.8799]	0.0614 [0.0582, 0.0645]	0.7176 [0.7036, 0.7317]	0.1259 [0.1199, 0.1320]
	**After tuning**	0.9330 [0.9287, 0.9374]	0.0421 [0.0397, 0.0445]	***0.9445*** ***[0.9408, 0.9481]***	***0.0379*** ***[0.0356, 0.0403]***	***0.8631*** ***[0.8553, 0.8710]***	***0.0831*** ***[0.0783, 0.0879]***
**Random Forest**	**Before tuning**	0.8758 [0.8690, 0.8825]	0.0591 [0.0563, 0.0618]	0.8437 [0.8361, 0.8513]	0.0684 [0.0650, 0.0717]	0.6924 [0.6770, 0.7077]	0.1288 [0.1228, 0.1348]
	**After tuning**	0.9348 [0.9308, 0.9388]	0.0401 [0.0380, 0.0421]	0.9349 [0.9309, 0.9388]	0.0431 [0.0408, 0.0454]	0.8196 [0.8102, 0.8289]	0.1000 [0.0956, 0.1044]
**Decision Tree**	**Before tuning**	0.7850 [0.7770, 0.7931]	0.0794 [0.0761, 0.0826]	0.7415 [0.7325, 0.7504]	0.0835 [0.0801, 0.0869]	0.5962 [0.5840, 0.6083]	0.1285 [0.1232, 0.1339]
	**After tuning**	0.8745 [0.8682, 0.8807]	0.0609 [0.0583, 0.0636]	0.8828 [0.8764, 0.8892]	0.0611 [0.0585, 0.0637]	0.6991 [0.6842, 0.7139]	0.1331 [0.1278, 0.1384]
**Extra Trees**	**Before tuning**	0.8739 [0.8668, 0.8811]	0.0591 [0.0564, 0.0618]	0.8438 [0.8357, 0.8520]	0.0686 [0.0647, 0.0725]	0.6901 [0.6747, 0.7055]	0.1300 [0.1241, 0.1359]
	**After tuning**	0.9386 [0.9344, 0.9428]	0.0403 [0.0383, 0.0423]	0.9420 [0.9382, 0.9458]	0.0393 [0.0371, 0.0415]	0.8424 [0.8323, 0.8526]	0.0879 [0.0824, 0.0934]
**Gradient Boosting**	**Before tuning**	0.8825 [0.8764, 0.8886]	0.0572 [0.0547, 0.0598]	0.8550 [0.8473, 0.8628]	0.0655 [0.0618, 0.0691]	0.6910 [0.6746, 0.7073]	0.1310 [0.1250, 0.1369]
	**After tuning**	0.9316 [0.9277, 0.9355]	0.0427 [0.0403, 0.0451]	0.9354 [0.9312, 0.9396]	0.0423 [0.0401, 0.0446]	0.8151 [0.8062, 0.8240]	0.1034 [0.0976, 0.1092]

**Table 3:** Summary of the prediction performance on the hold-out test data before the pipeline tuning (basic radiomics pipeline) and after the pipeline tuning (final radiomics pipeline). The mean and standard deviation (in brackets) AUC/accuracy averaged on the 51 random seeds are reported, regarding different tumor grades, IDH mutation status, 1p/19q codeletion status, and the glioma subtypes. Note that, when reporting the accuracy, the threshold that maximizes the F1-score of the train data were chosen as the final threshold for each binary classification task.

	Tumor Grade		IDH Mutation Status		1p/19q Codeletion Status		5 Glioma Subtypes
	AUC	Accuracy	AUC	Accuracy	AUC	Accuracy	Accuracy
**Basic radiomics pipeline** (Section 2.4)	0.8935 (±0.0351)	0.8171 (±0.0442)	0.8676 (±0.0421)	0.7840 (±0.0636)	0.6473 (±0.1074)	0.7209 (±0.2250)	0.5772 (±0.0816)
**Final radiomics pipeline** (Section 3.2)	0.9319 (±0.0386)	0.8453 (±0.0691)	0.9283 (±0.0333)	0.8560 (±0.0333)	0.8196 (±0.0702)	0.8499 (±0.0351)	0.6716 (±0.0655)

## Data Availability

The data used in this paper are public available. Researchers can download the clinical MRI images of the dataset from The Cancer Imaging Archive (TCIA) platform, with the link https://wiki.cancerimagingarchive.net/pages/viewpage.action?pageId=24282666 (accessed on 18 July 2021) for the TCGA-GBM dataset and the link https://wiki.cancerimagingarchive.net/pages/viewpage.action?pageId=24282668 (accessed on 18 July 2021) for the TCGA-LGG dataset. The corresponding gene status and clinical information can be downloaded from The Cancer Genome Atlas Program (TCGA) platform using TCGAbiolinks and R codes, see our codes here https://github.com/Yingping-LI/Radiomics/tree/main/extract_gene_data, accessed on 29 March 2022.

## Abbreviations

The following abbreviations are used in this manuscript:

CNS	central nervous system
WHO	World Health Organization
2016 CNS WHO	2016 World Health Organization Classification of Tumors of the
	Central Nervous System
TCGA	The Cancer Genome Atlas
TCIA	The Cancer Imaging Archive
LGG	low-grade glioma
GBM	glioblastoma multiforme
TCGA-LGG	The Cancer Genome Atlas Low Grade Glioma
TCGA-GBM	The Cancer Genome Atlas Glioblastoma Multiforme
IDH	isocitrate dehydrogenase
MGMT	O6-methylguanine-DNA methyltransferase
MRI	magnetic resonance imaging
FLAIR	fluid attenuated inversion recovery
VOI	volume of interest
ROC AUC	area under the receiver operating characteristic curve
ROC	receiver operating characteristic
AUC	area under the ROC curve
EB	empirical Bayes
BraTS	brain tumor segmentation
CI	confidence interval
SVM	support vector machine
GLRLM	gray level run length matrix
GLSZM	gray level size zone matrix
GLDM	gray level dependence matrix
NGTDM	neighbouring gray tone difference matrix
GLCM	gray level co-occurrence matrix

## Appendix A

### Appendix A.1. Summary of the Train and Test Data

In total, 212 patients from the retrospective TCGA-GBM and TCGA-LGG datasets were studied in this paper. The 212 patients were split into the train and test data randomly, at a 7:3 ratio, in a stratified fashion (stratified by the five tumor subtypes). The sizes of the train and test data regarding different glioma subtypes, and regarding tumor grade, IDH mutation status, and 1p/19q codeletion status, are summarized in Table A1 and Table A2, respectively.

**Table A1:** Summary of the train and test data regarding different glioma subtypes.

Tumor Subtypes	Train Data	Test Data	Total
1—LGG, IDH mutant, 1p/19q codeleted	19	8	27
2—LGG, IDH mutant, 1p/19q intact	40	17	57
3—LGG, IDH wild type	14	7	21
4—GBM, IDH mutant	3	1	4
5—GBM, IDH wild type	72	31	103

**Table A2:** Summary of the data regarding different tumor grades, IDH mutation status, and 1p/19q codeletion status.

Data	Tumor Grade	IDH Mutation	1p/19q Codeletion
Train data (148 patients)	75 GBM vs. 73 LGG	62 IDH mutant vs. 86 IDH wild type	129 1p/19q codeleted vs. 19 1p/19q intact
Test data (64 patients)	32 GBM vs. 32 LGG	26 IDH mutant vs. 38 IDH wild type	56 1p/19q codeleted vs. 8 1p/19q intact

### Appendix A.2. Image Histograms before and after Normalization

Figure A1 visualizes the image histograms of the MRI images in our study before and after Z-score intensity normalization.
